# Apigenin, a potent suppressor of dendritic cell maturation and migration, protects against collagen‐induced arthritis

**DOI:** 10.1111/jcmm.12717

**Published:** 2015-10-30

**Authors:** Xing Li, Yanping Han, Qingyou Zhou, Hongyu Jie, Yi He, Jiaochan Han, Juan He, Yong Jiang, Erwei Sun

**Affiliations:** ^1^ Department of Rheumatology and ImmunologyThe Third Affiliated Hospital of Southern Medical UniversityGuangzhouChina; ^2^ Institute of Clinical ImmunologyAcademy of Orthopedics, Guangdong ProvinceChina; ^3^ Hospital of South China Normal UniversityGuangzhouChina; ^4^ Key Laboratory of Proteomics of Guangdong ProvinceDepartment of PathophysiologySouthern Medical UniversityGuangzhouChina; ^5^ State Key Laboratory of Organ Failure ResearchNanfang HospitalSouthern Medical UniversityGuangzhouChina

**Keywords:** rheumatoid arthritis, dendritic cells, apigenin, maturation, migration

## Abstract

This study aimed to investigate whether apigenin (API) suppresses arthritis development through the modulation of dendritic cell functions. Bone marrow‐derived dendritic cells (BMDCs) were stimulated *in vitro* with lipopolysaccharide (LPS) and treated with API for 24 hrs; DC functions, including phenotype expressions, cytokine secretion, phagocytosis and chemotaxis, were then investigated. The effects of API on collagen‐induced arthritis (CIA) were examined *in vivo*, and purified DCs from the lymph nodes (LNs) of API‐treated CIA mice were analysed for phenotypes and subsets. In *in vitro*, API efficiently restrained the phenotypic and functional maturation of LPS‐stimulated BMDCs while maintaining phagocytotic capabilities. Moreover, API inhibited the chemotactic responses of LPS‐stimulated BMDCs, which may be related to the depressive effect on chemokine receptor 4 (CXCR4). In *in vivo*, API treatment delayed the onset and reduced the severity of arthritis in CIA mice, and diminished secretion of pro‐inflammatory cytokines in the serum and supernatants from the LN cells of the CIA mice. Similar to the *in vitro* findings, the API‐treated mice exhibited reduced expression of co‐stimulatory molecules and major histocompatibility complex II on DCs. Furthermore, API treatment strongly down‐regulated the number of Langerhans cells, but not plasmacytoid DCs (pDCs) in LNs, which may be related to the depressive effect of API on the expression of CXCR4 on DCs of peripheral blood. These data provide new insight into the mechanism of action of API on arthritis and indicate that the inhibition of maturation and migration of DCs by API may contribute to its immunosuppressive effects.

## Introduction

Rheumatoid arthritis (RA) is a chronic autoimmune disorder that is characterized by systemic and joint synovial chronic inflammation and/or by bone and cartilage destruction 1. Despite extensive efforts, the molecular pathogenesis and aetiological factors of RA are not entirely obvious, and effective treatments with limited side‐effects remain lacking. We are now beginning to understand that the functions of dendritic cells (DCs), especially maturation and migration, are important constituents in the pathogenesis of RA.

Dendritic cells, the professional antigen‐presenting cells, are specialized cells that acquire and process protein antigens, assemble antigenic peptides on the major histocompatibility complex (MHC) and present them to T cells. DCs are critical sentinels to induce and regulate most adaptive immune responses 2, 3. Dendritic cells play central roles in linking innate and adaptive immunities, which likely contribute to the pathogenesis of RA in several ways: (*i*) Immature DCs infiltrate the synovial tissues and fluids of the involved joints in RA patients and mature within the inflammatory microenvironment 4; (*ii*) In the presence of chemotactic ligands, mature DCs rapidly transit through the endothelium of lymphatic vessels and migrate to the draining secondary lymphoid organs, where they present antigens to naïve T cells and induce T‐cell activation^5^; (*iii*) Pro‐inflammatory cytokines, such as tumor necrosis factor (TNF)‐α, interleukin (IL)‐1β and IL‐6, induce DC maturation and initiate pro‐inflammatory cytokine production (IL‐12p70), T‐cell‐mediated cytotoxity and B‐cell‐mediated antibody production 6. Taken together, the functions of DCs, particularly maturation and migration, play critical roles in the initiation and development of RA 7.

Apigenin (API, 4, 5, 7‐trihydroxy flavone, molecular formula C_15_H_10_O_5_), one of the bioactive components in plant flavones, has gained particular interest because of its strong anti‐inflammatory activities and potential roles in the prevention of cancers 8. Accumulating evidence has indicated that API induces anti‐inflammatory and immunomodulatory effects on macrophages 9 and monocytes 10. API also suppresses the occurrence and development of autoimmune diseases, such as lupus 11 and asthma 12. However, its effects on DCs, and especially, its clinical significance in RA remain unknown. Here, we investigated the immune regulatory effects of API on RA and primarily focused on DC functions.

## Materials and methods

### Animals

Eight‐ to 12‐week‐old C57BL/6 male mice and 6‐ to 8‐week‐old DBA/1J male mice were used for the experiments. Further details are described in Data S1.

### Phenotype and cytokine analyses

Bone marrow‐derived dendritic cells were generated *in vitro* from bone marrow according to the procedure described by Inaba *et al*., with modifications (for details see Data S1) 13. Then bone marrow‐derived dendritic cells (BMDCs) were treated with lipopolysaccharide (LPS; 1 μg/ml, *Escherichia coli* 0111:B4; Sigma‐Aldrich, St. Louis, MO, USA) in the presence of API (dissolved in dimethylsulfoxide (DMSO) at 20 μM; Sigma‐Aldrich) for 24 hrs. The cells were collected and stained with the following monoclonal antibodies for 30 min. at 4°C for phenotype analyses of DCs: CD11c (HL3), CD40 (3/23), CD80 (16‐10A1), CD86 (GL1) and I‐A^b^ β‐chain (C57BL/6 MHC II, AF6‐120.1) (all antibodies obtained from BD Pharmingen, San Diego, CA, USA). Flow cytometric analysis was performed on a FACScan equipped with Cell Quest software (Becton Dickinson, Heidelberg, Germany). For cytokine measurements, DCs were purified with anti‐CD11c‐coated magnetic beads (Miltenyi Biotech, Teterow, Köln, Germany). The purified BMDCs were treated with LPS (1 μg/ml) in the presence of API (20 μM). The supernatants were collected after 24 hrs. The cytokines, including TNF‐α, IL‐10, IL‐1β, IL‐6, IL‐17A and interferon(IFN‐γ) were measured with Bio‐Plex kit (Bio‐Rad Laboratories, Hercules, CA, USA) and analysed with the Bio‐Plex manager software (version 4.0) and The IL‐12p70 were measured with ELISA kits (R&D Systems, Minneapolis, MN, USA).

### Phagocytosis and chemotaxis assay

The phagocytosis by BMDCs was determined by dextran‐FITC (Sigma‐Aldrich) uptake, as previously described 14. Further details are described in Data S1. An assay for BMDC migration in response to the chemokine CCL21 was performed with 24‐well transwell chambers (8.0 μm pore size; Corning, Acton, MA, USA), as previously described 15. Further details are given in Data S1.

### CFSE labelling and *in vivo* monitoring of BMDCs

The purified BMDCs generated from bone marrow were treated with LPS (1 μg/ml) for 24 hrs. Then the DCs were incubated in pre‐warmed PBS that contained carboxyfluorescein diacetatesuccinimidyl ester (CFSE, 5 μM; Invitrogen, Carlsbad, CA, USA) for 15 min. at 37°C; the labelled BMDCs (1 × 10^6^) were injected subcutaneously into the footpads of the mice. In the API treatment group, the mice were administered 20 mg/kg API intraperitoneally 2 hrs prior to cell injection. The control group was injected with the same amount of vehicle solution (DMSO‐PBS). Following an additional 24 hrs, the lymph node (LN) cells from the draining popliteal LNs were collected, and flow cytometry was used to analyse the CFSE^+^‐migrated cells.

### Chemokine receptor analyses

The BMDCs were treated with LPS (1 μg/ml) for 24 hrs and were then incubated with API (20 μM) for an additional 5 hrs. The cells were collected and stained with the following monoclonal antibodies for 30 min. at 4°C for chemokine receptor analyses of DCs: chemokine receptor 4 (CXCR4), CCR7 and CCR5 (BD Pharmingen).

### Collagen‐induced arthritis model and histology

Collagen‐induced arthritis (CIA) was induced in DBA/1J mice as previously described 16. Apigenin was administered intraperitoneally (20 mg/kg) at the time of the first collagen injection, with treatment continued daily until the end of the experiment 11. The mice were carefully monitored for the development and severity of joint inflammation and were scored by two trained examiners *via* a visual assessment scoring system 17. For histology, the joints of the mice were removed, fixed in formalin, decalcified in 10% ethylenediaminetetraacetic acid for 14 days and embedded in paraffin. The sections were stained with haematoxylin and eosin, and the score blinded for signs of arthritis. Further details are given in Data S1.

### Cytokine analysis

For the *in vivo* cytokine analyses, the sera were collected from the blood of all mice at day 48; the cytokines (TNF‐α, IL‐1β and IL‐6) were then measured using ELISA kits (R&D Systems). For the analysis of cytokine production by LN cells, the cells (1 × 10^6^) from LNs were cultured in triplicate in 24‐well plates that contained 50 μg/ml bovine collagen II (Col II) for 3 days at 37°C in 5% CO_2_. Next, the supernatants were collected and the cytokine levels were measured by ELISA.

### Analysis of DC phenotypes and subsets in LNs

On day 48, peripheral blood mononuclear and LN cells were collected and counted. Further details are described in Data S1. The CD11c^+^ cells were harvested and analysed by flow cytometry; then, single‐cell suspensions were prepared from the LNs of healthy and API‐ or vehicle‐treated CIA mice. The DCs were purified with anti‐CD11c‐coated magnetic beads and stained with the following monoclonal antibodies for 30 min. at 4°C: CD40, CD80, CD86, I‐Aq (DBA/1J MHC II) for the analysis of DC maturation and CD11c, Langerin (4CF), PDCA‐1(927) and B220 (RA3‐6B2) (Biolegend, San Diego, CA, USA) for the analysis of DC subsets.

### Analysis of chemokine receptors on DCs of peripheral blood

The DCs in peripheral blood mononuclear cells of all mice were harvested and flow cytometry was used to analyse the expression of chemokine receptors, including CXCR4, CCR7 and CCR5.

### Statistical analysis

Statistical analysis was performed with GraphPad Prism software (San Diego, CA, USA). Intergroup comparisons were conducted using one‐way anova and the Least Significant Difference (LSD) test. Bonferroni's analysis was used to compare all pairs of means following the one‐way anova. A value of *P* < 0.05 was considered significant.

## Results

### API efficiently inhibited DC maturation and reduced cytokine secretion while maintaining phagocytotic capabilities

**Figure 1:**
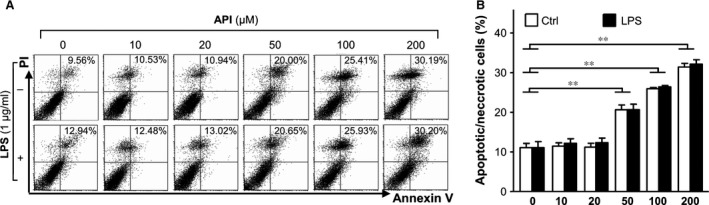
Evaluation of the cytotoxicity of apigenin (API) on BMDCs. (**A** and **B**) BMDCs were generated from bone marrow of C57BL/6 mice and purified with anti‐CD11c‐coated magnetic beads using the autoMACS system. Purified BMDCs (>95% for CD11c^+^ cells) were treated with various concentrations of API in the absence or presence of LPS for 24 hrs, and then stained with Annexin V‐FITC/Propidium Iodine Apoptosis Detection Kit (Invitrogen, Carlsbad, CA, USA). Apoptotic and necrotic rates were determined by flow cytometry. The values are means ± SEM of samples from three wells. **P < 0.01 were for the comparisons between API‐treated and LPS‐ induced DCs *versus* API 0 μM.

Prior to the beginning of this study, it was essential to determine how well DCs could tolerate API treatment. Thus, we determined the cytotoxicity of API on mouse BMDCs. Our data indicated that API induced significant BMDC apoptosis and necrosis only at concentrations of 50 μM or more, but not at concentrations of 20 μM or less (Fig. 1A and B). Therefore, 20 μM API was chosen for the subsequent experiments.

**Figure 2:**
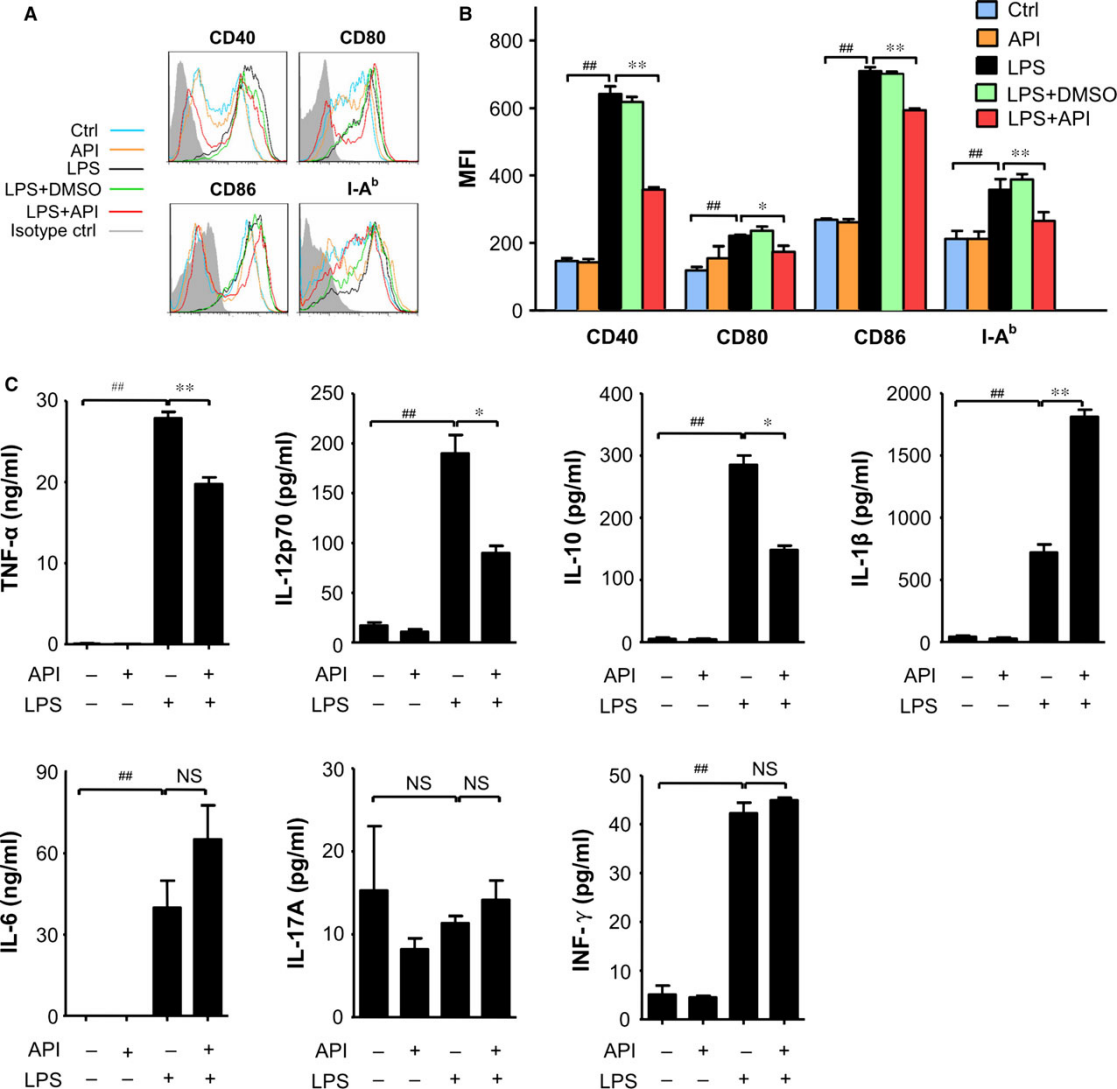
API efficiently inhibited the LPS‐induced DC maturation. Bone marrow‐derived DCs (BMDCs) were stimulated with medium, API (20 μM), LPS, LPS+DMSO (0.2%) or LPS+API (20 μM) for 24 hrs. The expression levels of CD40, CD80, CD86 and MHC II (I‐A^b^) were determined by flow cytometry. All data shown were gated on CD11c^+^cells. (**A**) The histogram of CD40, CD80, CD86 and MHC II (I‐A^b^) in the CD11c^+^ cells are shown, with the grey‐filled area representing staining with isotype control Abs. The histogram was obtained from a single experiment that is representative of three independent experiments. (**B**) The changes in mean fluorescence intensity (MFI) of CD40, CD80, CD86 and MHC II (I‐A^b^) in the CD11c^+^ cells were analysed. (**C**) BMDCs were stimulated with medium, API (20 μM), LPS+DMSO (0.2%), or LPS+API (20 μM) for 24 hrs. The expression of cytokines, including TNF‐α, IL‐10, IL‐1β, IL‐6, IL‐17A and INF‐γ were measured with Bio‐Plex kit and analysed with the Bio‐Plex manager software and IL‐12p70 were measured with ELISA kits. The values represent the mean ± SEM of the samples from three wells. NS,* P* > 0.05; ^#^ *P* < 0.05; ^##^ *P* < 0.01 were for the comparisons between the LPS‐treated and ‐untreated DCs; *P < 0.05; **P < 0.01 were for the comparisons between the API‐treated and ‐untreated LPS‐induced DCs. All data are representative of three independent experiments.

**Figure 3:**
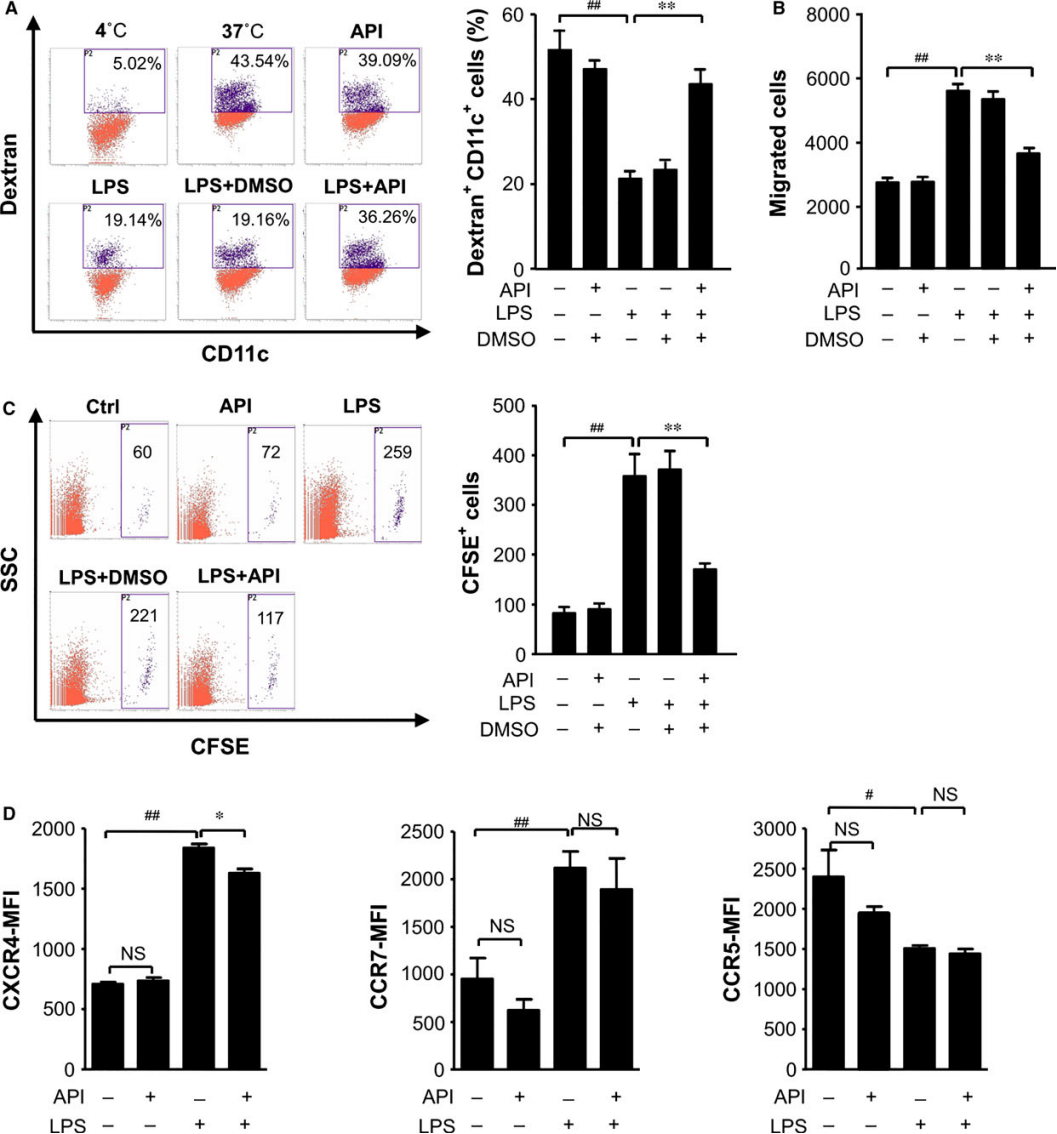
API maintained high phagocytotic capabilities and reduced the migration of LPS‐treated BMDCs. The BMDCs were treated with LPS (1 μg/ml) in the presence of API (20 μM) or 0.4% DMSO for 24 hrs. (**A**) Phagocytosis was determined following the exposure of BMDC to FITC‐dextran at 4 and 37°C for 45 min. All data shown were gated on the CD11c^+^ cells. The percentage of dextran‐FITC ^+^ CD11c^+^ cells is indicated. The cells incubated with dextran‐FITC at 4°C served as the negative control; the cells stored at 37°C were used as the non‐treatment control. (**B**) The assay for BMDC migration in response to CCL21 was performed in 24‐well transwell chambers. The migrated DCs in the bottom chambers were harvested and counted with flow cytometry. (**C**) CFSE labelling and *in vivo* monitoring of the migration of the BMDCs. Flow cytometry was used to analyse the CFSE ^+^ migrated cells. (**D**) Effects of API treatment on CXCR4,CCR7 and CCR5 expression by LPS‐stimulated DCs. For cells labelled with mAb or isotype IgG, the CXCR4, CCR7 and CCR5 expression was analysed by flow cytometry. The changes in mean fluorescence intensity (MFI) of CXCR4, CCR7 and CCR5 in the CD11c^+^ cells were analysed. The values represent the mean ± SEM of samples from three wells (**A**,** B** and **D**) or three mice (**C**). NS,* P* > 0.05; ^#^ *P* < 0.05; ^##^ *P* < 0.01 were for the comparisons between the LPS‐treated and ‐untreated DCs. *P < 0.05; **P < 0.01 were for the comparisons between the API‐treated and ‐untreated LPS‐stimulated DCs.

Next, we used GM‐CSF‐treated BMDCs to test the effects of API on the maturation of DCs. The BMDCs were resuspended and treated with 20 μM API in the presence or absence of LPS (1 μg/ml) for an additional 24 hrs. As shown in Figure 2A and B, API markedly suppressed the expression of co‐stimulatory molecules, such as CD40, CD80, CD86 and MHC II (I‐A^b^), on the surfaces of the CD11c^+^cells. Furthermore, API inhibited the capacity of LPS‐stimulated BMDCs to produce cytokines including TNF‐α, IL‐12p70 and IL‐10 without a significant effect on IL‐6, IL‐17A and IFN‐γ, while increased the secretion of IL‐1β (Fig. 2C). Therefore, API is a potent inhibitor of DC maturation by reducing both maturation marker expression and cytokine production. To the best of our knowledge, DC maturation is typically accompanied by diminished phagocytotic capability. To determine the effects of API on DC phagocytosis, we examined the intake of FITC‐conjugated dextran by BMDCs. Following the incubation of the BMDCs with LPS for 24 hrs, the intake of dextran‐FITC was profoundly inhibited, whereas API treatment dramatically resisted the inhibitory effect of LPS (Fig. 3A). These results excluded the possibility that down‐regulation of DC maturation markers were the result of DC cytotoxicity.

### API inhibited the migration of LPS‐treated BMDCs and had depressive effect on chemokine receptor 4 (CXCR4)

The migration of DCs is critical and indispensable for immune surveillance 18. To understand the effects of API on DC migration, we performed a migration assay with transwell chambers. As shown in Figure 3B, the LPS‐stimulated BMDCs responded to CCL21 and efficiently migrated from the top wells to the bottom wells. However, API‐treated BMDCs markedly reduced the migration in response to CCL21. Next, we investigated the effects of API on DC migration *in vivo*. The LPS‐stimulated BMDCs were labelled with CFSE and injected subcutaneously into the footpads of mice. Following an additional 24 hrs, the labelled DCs collected from the draining LNs were counted using flow cytometry. The results indicated that the migration of the LPS‐stimulated BMDCs *in vivo* was markedly down‐regulated by API treatment (Fig. 3C). To investigate the mechanisms underlying this inhibition of DC migration by API, we detected the expression of chemokine receptors on LPS‐stimulated DCs. As shown in Figure 3D, LPS‐stimulated DCs expressed elevated levels of CXCR4 and CCR7,but decreased levels of CCR5 compared with untreated DCs. However, the API treatment of LPS‐stimulated DCs down‐regulated the expression of CXCR4, but not CCR7 and CCR5 (Fig. 3D).

### API treatment delayed the onset and reduced the severity of joint arthritis in a CIA model

**Figure 4:**
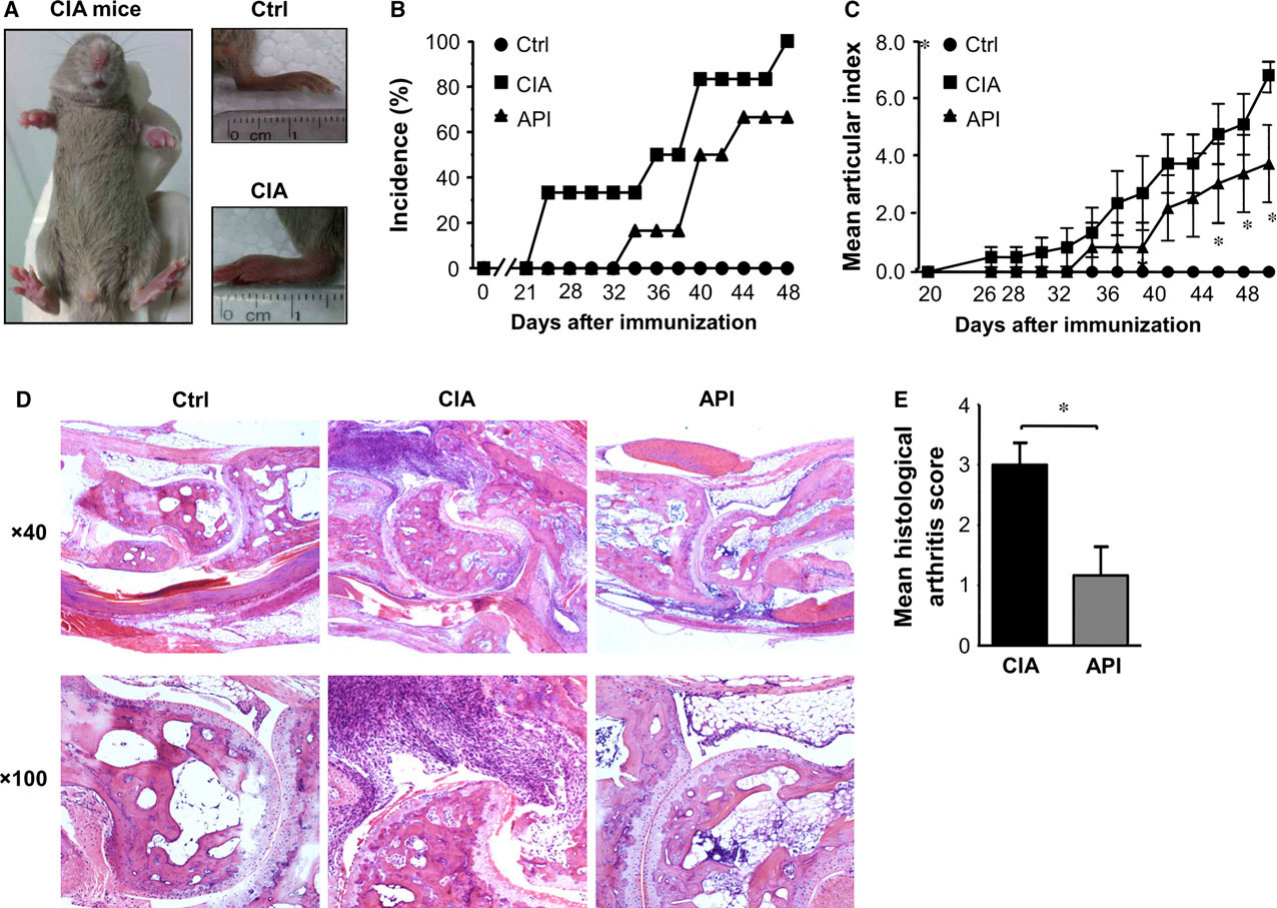
API treatment delayed the onset of CIA and reduced the severity of CIA. (**A**) The CIA model in DBA/1J mice was successfully generated. (**B**) The API‐treated CIA mice showed a significant decrease in disease incidence compared with the vehicle‐treated CIA mice. (**C**) The severity of arthritis was assessed using a visual arthritis scoring system. The API‐treated CIA mice showed a dramatic decrease in arthritis scores compared with the vehicle‐treated CIA mice. (**D** and **E**) Histological arthritis score of haematoxylin and eosin‐stained inflamed joints following vehicle or API administration. Values represent the mean ± SEM (*n* = 6). *P < 0.05 were for the comparisons between the API‐treated and vehicle‐treated CIA mice.

*In vitro* experiments have previously demonstrated that API treatment could modulate the functions of BMDCs. Because DCs play key roles in the pathogenesis of RA, we investigated whether API treatment would have beneficial effects on disease courses in a mouse CIA model that exhibited many clinical and histological features similar to RA (Fig. 3A). Our data demonstrated that compared with the vehicle‐treated CIA mice, the API‐treated CIA mice showed a delayed onset of CIA, a decreased incidence of arthritis and reduced joint swelling as determined by clinical scores (Fig. 4B and C). Histological examinations also indicated that API treatment suppressed synovial hyperplasia and the accumulation of inflammatory cells in the joint space (Fig. 4D and E).

### API treatment reduced cytokine levels in the serum and the supernatant from the cells of LNs in CIA mice

**Figure 5:**
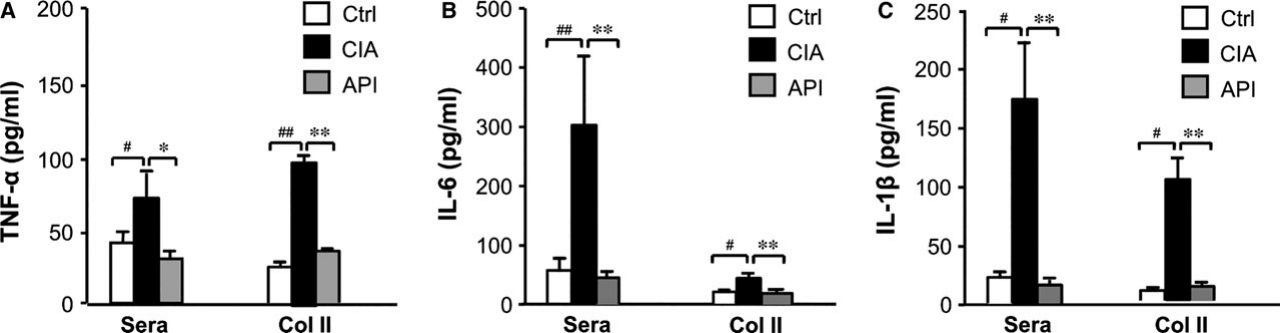
Analysis of the cytokine levels of sera and cultured primary cells of LNs. At the end of the experiment, the sera and supernatant from the *ex‐vivo* cultured LN cells of the CIA mice were collected, and the cytokine levels were measured by ELISA using the manufacturer's protocols. For the analysis of cytokine production by LN cells, cells (1 × 10^6^)from LNs were cultured in triplicates in 24‐well plates containing bovine Col II (50 μg/ml) for 3 days at 37°C in 5% CO_2_ for 3 days. (**A**–**C**, API treatment profoundly inhibited the production of the pro‐inflammatory cytokines TNF‐α, IL‐1β and IL‐6 in serum and the *ex‐vivo* cultured LN cells of the CIA mice. Values represent the mean ± SEM (*n* = 6). ^#^ *P* < 0.05; ^##^ *P* < 0.01 were for the comparisons between the healthy control mice and the vehicle‐treated CIA mice. *P < 0.05;**P < 0.01 were for the comparisons between the API‐treated and vehicle‐treated CIA mice.

The levels of the pro‐inflammatory cytokines TNF‐α, IL‐1β and IL‐6 in serum were measured by ELISA at the termination of the experiment. Consistent with joint swelling, the serum levels of TNF‐α, IL‐1β and IL‐6 in the vehicle‐treated CIA mice were significantly increased, whereas API treatment exhibited a dramatic decrease in these cytokines (Fig. 5A–C). We also investigated the levels of these cytokines in bovine Col II stimulated cells that were isolated from the LNs of the CIA mice. Similarly, API treatment significantly decreased the production of the pro‐inflammatory cytokines TNF‐α, IL‐1β and IL‐6 in the supernatant from the LN cells of the CIA‐mice (Fig. 5A–C).

### API treatment suppressed DC maturation during the acute phase of CIA

**Figure 6:**
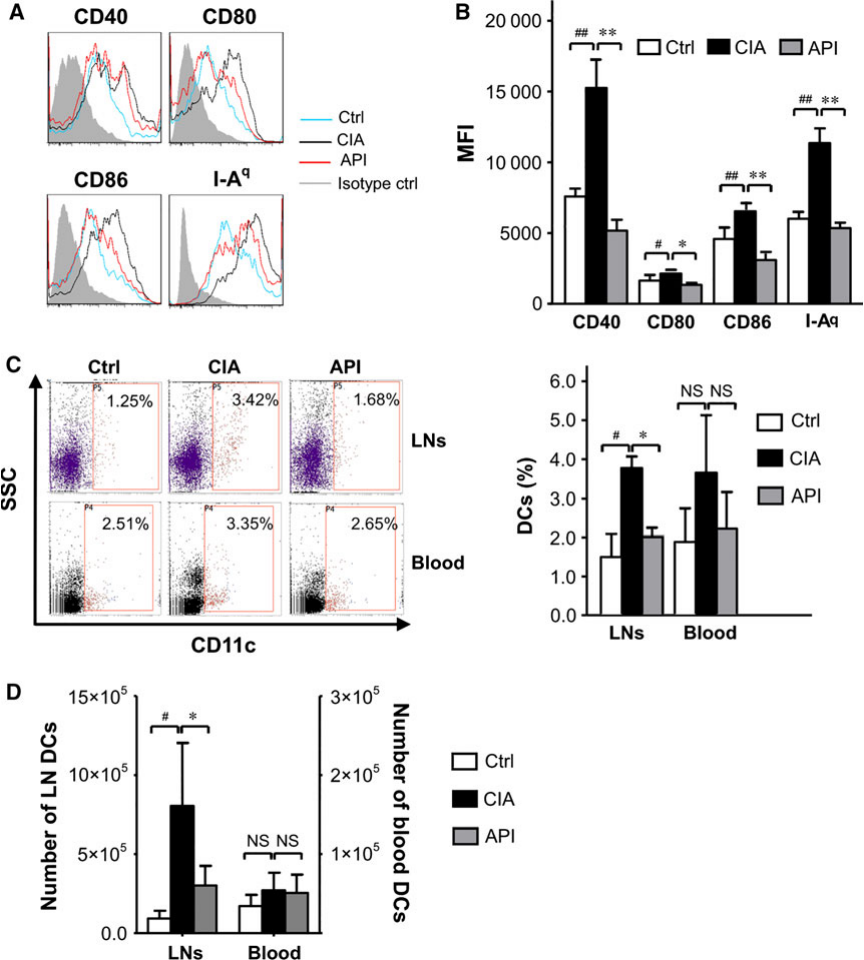
API treatment suppressed DC maturation and reduced the percentages of DCs in LNs during the acute phase of CIA. Single‐cell suspensions were prepared from the LNs of healthy and API‐ or vehicle‐treated CIA mice. The DCs were purified with anti‐CD11c‐coated magnetic beads. (**A**) The histograms of CD40, CD80, CD86 and MHC II (I‐A^q^) in the CD11c^+^ cells are shown; the grey‐filled areas represent staining with an isotype‐matched control Ab stain. (**B**) The changes of mean fluorescence intensity of CD40, CD80, CD86 and MHC II (I‐A^q^) were analysed. (**C** and **D**) API treatment reduced the percentages and aboslute numbers of DCs in LNs, but not in the blood of the CIA mice. Values represent the mean ± SEM (*n* = 6). NS,* P* > 0.05; ^#^ *P* < 0.05; ^##^ *P* < 0.01 were for the comparisons between the healthy control mice and the vehicle‐treated CIA mice. *P < 0.05; **P < 0.01 were for the comparisons between the API‐treated and vehicle‐treated CIA mice.

To study the effect of API treatment on DC maturation in the CIA model, all cells from the bilateral inguinal, the popliteal fossa, the tail and the axillary LNs were harvested and purified by immunomagnetic sorting with anti‐CD11c‐coated magnetic beads. Next, the cells were stained for cell membrane markers on the DCs. The results demonstrated a significant increase in the expressions of CD40, CD80, CD86 and MHC II (I‐A^q^) on the surfaces of the CD11c^+^cells in the CIA mice compared with the healthy control mice. However, API treatment significantly inhibited the maturation of DCs in the LNs of the CIA mice (Fig. 6A and B).

### API treatment reduced the percentages of DCs in the LNs and changed DC subsets of LNs

To assess the effect of API treatment on DC migration in the CIA model, we first investigated the effect of API on DC distribution in peripheral blood and draining LNs. Our data showed that API treatment dramatically decreased the percentages and absolute number of DCs in the LNs compared with that in CIA mice. However, there were no significant differences in the number of DCs in peripheral blood between those two groups (Fig. 6C and D).

**Figure 7:**
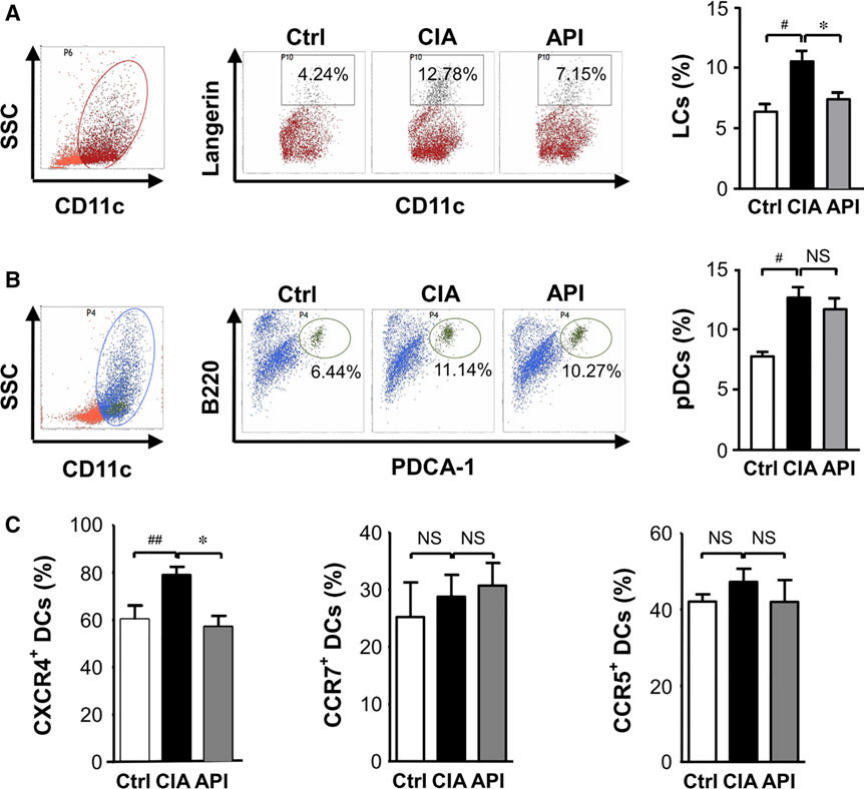
API treatment changed the DC subsets of LNs in CIA mice and down‐regulated the CXCR4 expression on blood DCs. Single‐cell suspensions were prepared from the LNs of healthy and API‐ or vehicle‐treated CIA mice. The DCs were purified with anti‐CD11c‐coated magnetic beads and stained with the following monoclonal antibodies for 30 min. at 4°C: CD11c, Langerin, PDCA‐1, and B220 for the analysis of DC subsets. (**A**) API treatment significantly reduced the number of LCs (Langerin^+^ CD11c^+^)in the LNs. (**B**) There were no differences in the number of pDCs(B220^+^ PDCA‐1^+^ CD11c^+^) between the API‐ and vehicle‐treated groups. (**C**) Effects of API treatment on CXCR4, CCR7 and CCR5 expression on DCs of peripheral blood. Peripheral blood mononuclear cells were prepared from the blood of healthy, API‐ or vehicle‐treated CIA mice. The cells were labelled with mAb or isotype IgG, and the CXCR4, CCR7 and CCR5 expression in CD11c^+^ cells analysed by flow cytometry. Values represent the mean ± SEM (*n* = 6). NS,* P* > 0.05; ^#^ *P* < 0.05; ^##^ *P* < 0.01 was for the comparison between the healthy control mice and the vehicle‐treated CIA mice. *P < 0.05 was for the comparison between the API‐treated and vehicle‐treated CIA mice.

Next, the subsets of DCs in LNs involved in the development of CIA were assessed. The results demonstrated a significant increase in the percentages of Langerhans cells (LCs, langerin^+^ CD11c^+^DCs) and plasmacytoid DCs (pDCs, B220^+^ PDCA^+^ DCs) in LNs of CIA mice compared with that in LNs of the healthy control mice. The API treatment significantly reduced the number of LCs in the LNs of the CIA mice (Fig. 7A), whereas there was no difference in the number of pDCs between the API‐ and vehicle‐treated CIA mice (Fig. 7B).

### API treatment inhibited the expression of CXCR4 on DCs of peripheral blood

We then detected the expressions of chemokine receptors on the surfaces of DCs in peripheral blood in the CIA model. The results showed that, compared with the CIA mice, the API‐treated mice dramatically down‐regulated the expression of CXCR4, but not CCR7 and CCR5, on the surfaces of DCs in peripheral blood (Fig. 7C).

## Discussion

The central role of DCs in the induction of immunity is a significant component of RA pathogenesis 19. In RA, DCs become mature and up‐regulate the expression of MHC II and co‐stimulating molecules; they then migrate to the secondary lymphoid organs, where they present acquired antigens to naïve T lymphocytes and produce cytokines. Effective antigen presentation results in the differentiation of naïve T cells into T helper cells and promotes the maturation of antibody‐producing B cells 18, 20. Evidence has indicated that the synovial tissue of RA patients is enriched with DCs that are highly T‐cell stimulatory compared with normal synovia 21. Furthermore, animal research has shown that the transfer of collagen‐loaded DCs to joints is able to induce autoimmune arthritis 22. Therefore, DC functions, especially maturation and migration, remain potentially valuable targets for the control of RA. Here, we investigated whether a novel small molecule agent, API, could prevent RA development *via* the modulation of DC functions.

We first investigated the effects of API on the functional properties of murine BMDCs stimulated with LPS, a TLR4 ligand, *in vitro*. The TLR signals within DCs are critical for the orchestration of immune responses 23, 24. During the steady‐state, TLR ligands specifically recognize TLRs of DCs and activate mitogen‐activated protein kinase (MAPK), Nuclear factor‐κB (NF‐κB), and other intracellular signals to induce phenotypic and functional maturation 25, 26, 27. It has been reported that API targets multiple intracellular signals, such as the blockade of extracellular regulated protein kinases (ERK) and Jun N‐terminal kinase (JNK) phosphorylation to inhibit invasiveness and metastasis 28 and the suppression of TNF‐α‐induced prostaglandin E2 expression *via* the attenuation of the NF‐κB pathway 29. Our results indicate that API efficiently inhibits the phenotypic and functional maturation of DCs as indicated by the suppression of the expression of the co‐stimulatory molecules and MHC II and by a reduction in the secretion of cytokines, such as TNF‐α, IL‐12p70 and IL‐10. The effects of maturation inhibition by API on DCs may be related to its effects on the signal pathways of NF‐κB and MAPK. Because the mechanism of DC phagocytosis is a distinct characteristic of immature DCs, we investigated the capacity of API on the phagocytosis of DCs. The results suggest that API is only a potent inhibitor of DC maturation but not an enhancer of immature DC phagocytosis.

Migration is a crucial aspect of DC immunobiology 30. Accumulating evidence indicates that trafficking molecule matrix metalloproteinases (MMPs) 31, 32, chemokines 33 and adhesion molecules 34 play critical roles in the orchestration of DC migration. Once detachment from parenchymal tissues has been initiated by a mobilization signal, maturing DCs secrete MMPs, to disintegrate the extracellular matrix (ECM) barriers. Then, under the regulation of chemokines, and adhesion molecules, DCs travel to secondary lymphoid organs to activate T cells. Previous studies have indicated that API suppresses cellular invasion, adhesion and migration *via* the inhibition of enzymes implicated in ECM degradation, the modulation of adhesion molecule expression and the regulation of chemokine pathways 35, 36, 37, 38. Here, for the first time, we determined that the chemotactic response of LPS‐stimulated BMDC to chemokines was impaired following API treatment. Furthermore, the migration of LPS‐treated BMDC into draining LNs was also inhibited *in vivo* by API. To our knowledge, this is the first report that a small molecular agent can modulate DC migration both *in vitro* and *in vivo*. To further study the mechanisms underlying this inhibition of DC migration by API, we detected the expressions of chemokine receptors, including CXCR4, CCR7 and CCR5 on the surfaces of DCs. Immature DCs express CCR5. Upon exposure to maturation‐inducing stimuli, such as inflammatory cytokines and products of pathogens, DCs down‐regulate CCR5, and up‐regulate CCR7 and CXCR4 expression. Our results showed that DCs expressed elevated levels of CXCR4 and CCR7, but decreased levels of CCR5 after LPS stimulation. And following treatment with API, LPS‐stimulated DCs expressed persistent levels of CCR5 and CCR7 and failed to express elevated levels of CXCR4. This indicates that the mechanisms underlying the inhibition of DC migration to draining LNs by API might be at least partly because of the reduced expression of CXCR4. Nevertheless, the persistent expression of CCR7 following API could not explain why API hindered the migration of BMDC in response to its ligand, CCL21, *in vitro*. As has been previously described by Ren‐Yeong Huang, we speculate that the inhibition effects of API on the migration of BMDC in response to CCL21 may result from the effect of API on some other molecules downstream of CCR7, but not CCR7 itself 15, 39.

These findings provide new insights into the immunopharmacology of API and suggest a novel approach to the manipulation of DCs for the prevention and treatment of autoimmune diseases. Therefore, we investigated the effects of API on the pathogenesis of RA in a CIA model. We determined that API treatment markedly reduced clinical arthritis scores and the incidence of arthritis, as well as blocked synovial inflammation and joint destruction. The results indicate that API treatment not only prevents the onset of CIA but also suppresses the severity of the disease. The pro‐inflammatory cytokines TNF‐α, IL‐1β and IL‐6 are significant regulators in the pathogenesis of the immune‐mediated joint damage in the CIA model and RA patients 40, 41. To further elucidate the mechanisms involved in the protection of CIA by API, the production of the cytokines TNF‐α, IL‐1β and IL‐6 in the serum and supernatant from the LN cells of the CIA mice was investigated. As expected, treatment with API for 48 days resulted in a decrease in these cytokine levels in the sera and supernatants compared with CIA mice. These data suggest that API exerts strong anti‐inflammatory effects during the development of RA.

What are the mechanisms that underlie how API works in the CIA model? Consistent with the *in vitro* data, our results demonstrated that API treatment significantly reduced the expression of the co‐stimulatory molecules and MHC II on the surfaces of the DCs in the LNs of the CIA mice. These results suggest that the anti‐inflammatory activity exerted by API in RA may be, at least in part, because of the suppression of DC maturation. Moreover, API drastically down‐regulated the numbers of DCs in the LNs, but not in the blood of the CIA mice during CIA progression. These data may be interpreted as a depressive effect of API on DC migration into the LNs. The routes of DC migration into LNs differ notably for distinct DC subsets. Previous studies have reported that DC migration into LNs includes two pathways: lymph circulation and blood circulation. Langerhans cells, in which the DCs populate the epidermal layer of the skin, traffic into the LNs through afferent lymph flow, which is the same route as CD8α^+^DCs and conventional myeloid DCs. Plasmacytoid DCs appear incapable of migrating from the peripheral organs into the afferent lymph flow. They enter the LNs through high endothelial venules (HEVs) 39. Our data regarding the CFSE‐labelled BMDCs indicated that API treatment inhibited the migration of LPS‐treated BMDCs into draining LNs *in vivo*. To further study the effects of API on the routes of DC migration in RA, we detected the changes of LCs and pDCs in the LNs in the API‐treated and vehicle‐treated CIA mice. Our data revealed that the vehicle‐treated CIA mice showed a significant increase in the percentages of LCs and pDCs in LNs compared with the healthy control mice. Interestingly, API treatment only significantly reduced the number of LCs, but not pDCs in the LNs of the CIA mice. These findings indicate that API may specifically suppress LCs migration into the LNs *via* the afferent lymph vessels, but it may not suppress the pDCs *via* the HEVs. Previous studies have reported that CXCR4 inhibition can impair migration of LCs and dermal DCs to draining LNs. And our results also showed that API treatment dramatically down‐regulated the expression of CXCR4, but not CCR7 and CCR5 on DCs of peripheral blood in the CIA model, these are consistent with the *in vitro* findings.

In summary, our data clearly demonstrated that API suppresses DC maturation and migration, and highly efficaciously protects against inflammatory responses in a mouse CIA model. These results provide new insight into the mechanism of action of API on arthritis and indicate that the inhibition of maturation and migration of DCs by API may contribute to its immunosuppressive effects.

## Conflicts of interest

The authors confirm that there are no conflicts of interest.

## Author contribution

XL and YH designed the study; XL and YH performed the experiments and analysed the data; ES and YJ participated in the study with primary duties including the conception and design of the study, data analysis and interpretation of data, drafting the article and final proof of the revised manuscript. All authors substantially contributed to the interpretation of the data, critically revised the contents of the manuscript and approved the final version.

## Supporting information


**Data S1** Generation and purification of DCs from bone marrow (BMDCs).
