# Supplementary material for: Apigenin, a potent suppressor of dendritic cell maturation and migration, protects against collagen‐induced arthritis
Source: J Cell Mol Med. 2015 Oct 30;20(1):170–80. doi: 10.1111/jcmm.12717 (PMC4717846; doi:10.1111/jcmm.12717)
Supplement: Supplementary file 1 — Data S1 Generation and purification of DCs from bone marrow (BMDCs). [file JCMM-20-170-s001.doc]

**Supplementary Methods**

**Animals**

Male 8- to 12-week-old C57BL/6 (I-A^b^) were purchased from Department of Laboratory Animal Center of Southern Medical University (GuangZhou, China). Male 6- to 8-week-old DBA/1J (I-A^q^) mice were purchased from Shanghai Slac Laboratory Animal CO. LTD (ShangHai, China). They were housed and bred under specific pathogen-free(SPF) environment in the laboratory animal center of Sun Yat-sen University (Guangzhou, China). The animal experiments were conducted according to the ethical guidelines for animal experiments of Sun Yat-sen University and under experimental license approved by Guangdong1, China(NO.00047894 and NO. 00053305).

**Generation and purification of DCs from bone marrow (BMDCs)**

BMDCs were generated *in vitro* from bone marrow according to the procedure described by Inaba et al, with modifications [1]. Briefly, bone marrow cells (2×10^6^) of C57BL/6 mice were flushed out from the femur and tibiae using RPMI 1640 medium and passed through a cotton filter to remove bone marrow debris. They were then cultured at 37°C in 5% CO_2_ in 100-mm Petri-dishes that contained 10 ml RPMI 1640 medium supplemented with 10% heat-inactivated fetal bovine serum (FBS) (Atlanta Biological, NY), 2 mM L-glutamine, 100 U/ml penicillin, 100 μg/ml streptomycin, 5×10^-5^ M 2-ME and 20 ng/ml GM-CSF (PeproTech, Rocky Hill, NJ). On day 3, an additional 10 ml of complete medium that contained 20 ng/ml GM-CSF was added to each dish. On day 6, the floating cells were gently removed and fresh medium was replenished. On day 8, non-adherent cells and loosely adherent proliferating DCs were harvested and purified with anti-CD11c-coated magnetic beads using the autoMACS system (Miltenyi Biotech). The purity of the sorted cells was determined using flow cytometry (>95% for CD11c^+^cells).

**Phagocytosis assay.**

The phagocytosis by BMDCs was determined by dextran-FITC (Sigma-Aldrich, St. Louis, MO) uptake, as previously described [2]. In brief, the BMDCs were treated with LPS (1 μg/ml) in the presence of API (20 μM) for 24 h. Next, the cells (2×10^5^) were collected and equilibrated at 37°C or 4°C for 45 min and then incubated with dextran-FITC at a concentration of 1 mg/ml. Ice-cold staining buffer was pulsed to halt the reaction. The cells were washed three times with ice-cold phosphate buffered saline (PBS) that contained 0.1% NaN_3_ and stained with APC-conjugated anti-CD11c Abs. The percentage of the dextran-FITC^+^ CD11c^+^ population was analyzed with flow cytometry.

**Chemotaxis assay.**

An assay for BMDC migration in response to the chemokine CCL21 was performed using 24-well transwell chambers (8.0 μm pore size; Corning, Acton, MA), as previously described[3]. The bottom chambers of the transwell plates were filled with 500 μL serum-free medium with or without CCL21 (200 ng/ml, PeproTech, Rocky Hill, NJ). The BMDCs (2×10^5^) resuspended in serum-free medium were placed in the top chambers. Following incubation for 3 h at 37°C in 5% CO_2_, the DCs that had migrated in the bottom chambers were harvested and counted using flow cytometry.

**Induction and assessment of CIA**

Mice were immunized as previously described [4]. Briefly, Bovine type II collagen (CII) solution (Chondrex, Inc. USA) was dissolved in 0.05 M acetic acid to a concentration of 2.0 mg/ml by overnight rotation at 4°C and mixed with an equal volume of Freund's complete adjuvant (1mg/ml of Mycobacterium tuberculosis, Chondrex, Inc. USA). The mice were immunized subcutaneously at the base of the tail with 100 µl of emulsion on day 0. The booster injection(0.1ml CII emulsified with Freund's incomplete adjuvant) was repeated on day 21. Mice were inspected three times a week for signs of arthritis and were scored by two independent observers. The severity of the arthritis was assessed using an established semi-quantitative scoring system of 0–4 where 0=normal, 1=mild swelling, 2=moderate swelling, 3=swelling of all joints and 4=joint distortion and/or rigidity and dysfunction [5]. The cumulative score for all four paws of each mouse (maximum possible score 16) was used as the arthritis score to represent overall disease severity and progression in an animal. For the evaluation of incidence, mice were considered to have arthritis if the clinical arthritis score remained stable or increased by at least one point for two consecutive days.

**Histological analysis**

At the end of the CIA experiment, the mice were sacrificed, and joints of the mice were removed, fixed in formalin, decalcified in 10% Ethylene Diamine Tetraacetic Acid (EDTA) in H_2_O for 14 days, and paraffin embedded. Sections of 5 µm were stained with H&E and scored for histological signs of inflammation, pannus formation, and cartilage and bone destruction. Scores were as follows: 0: normal appearance; 1: mild inflammation and synovitis, mild cartilage destruction without bone erosion; 2–4: increasing degrees of inflammatory cell infiltration, synovitis and cartilage destruction.

**Analysis of the absolute numbers of DCs in blood and LNs.**

For cell counting in blood and LNs, we used the hemocytometer to count the cells. In brief, the equal volume of heparinized blood samples (200μl) were lysed using 2ml hypotonic ammonium chloride buffer. Inguinal and popliteal LNs were cut into small fragments and digested with 0.5mg/ml collagenase A (Roche, Germany) and 0.02mg/ml DNAse I (Roche, Germany) for 30 min at 37˚C with continuous agitation. Cell suspensions were made by filtering the digested fragments through a stainless steel screen. Then the total cells was counted with the hemocytometer under a microscope by two trained examiners for three times. Then the percent of CD11c+ cells were harvested and analyzed by flow cytometry, and the absolute numbers of DCs were calculated with the percent of DCs multiplied by the total cells.

**References**

1. **Inaba K, Inaba M, Romani N, et al.** Generation of large numbers of dendritic cells from mouse bone marrow cultures supplemented with granulocyte/macrophage colony-stimulating factor. *J Exp Med* 1992, 176:1693-702.

2. **Yu YL, Chen IH, Shen KY,et al.** A triterpenoid methyl antcinate K isolated from Antrodia cinnamomea promotes dendritic cell activation and Th2 differentiation. *Eur J Immunol.* 2009;39:2482-91.

3. **Huang RY, Yu YL, Cheng WC, et al.** Immunosuppressive effect of quercetin on dendritic cell activation and function. *J Immunol.* 2010;184(12):6815-21.

4. **Brand DD, Latham KA, Rosloniec EF.** Collagen-induced arthritis. *Nat Protoc.* 2007;2:1269-75.

5. **Sarkar S, Cooney LA, White P, et al.** Regulation of pathogenic IL-17 responses in collagen-induced arthritis: roles of endogenous interferon-gamma and IL-4. *Arthritis Res Ther.* 2009;11(5):R158.
